# Time-Based Decision Making for Reperfusion in Acute Ischemic Stroke

**DOI:** 10.3389/fneur.2021.728012

**Published:** 2021-11-01

**Authors:** Mathias Grøan, Johanna Ospel, Soffien Ajmi, Else Charlotte Sandset, Martin W. Kurz, Mona Skjelland, Rajiv Advani

**Affiliations:** ^1^Faculty of Medicine, University of Oslo, Oslo, Norway; ^2^Department of Radiology, Basel University Hospital, Basel, Switzerland; ^3^Department of Clinical Neurosciences, University of Calgary, Calgary, AB, Canada; ^4^Department of Neurology, Stavanger University Hospital, Stavanger, Norway; ^5^University of Stavanger, Stavanger, Norway; ^6^Stroke Unit, Department of Neurology, Oslo University Hospital, Oslo, Norway; ^7^Norwegian Air Ambulance Foundation, Oslo, Norway; ^8^Neuroscience Research Group, Stavanger University Hospital, Stavanger, Norway; ^9^Institute of Clinical Medicine, University of Oslo, Oslo, Norway

**Keywords:** stroke, thrombolysis, thrombectomy, acute ischaemia, magnetic resonance imaging (MRI), computer tomography (CT)

## Abstract

Decision making in the extended time windows for acute ischemic stroke can be a complex and time-consuming process. The process of making the clinical decision to treat has been compounded by the availability of different imaging modalities. In the setting of acute ischemic stroke, time is of the essence and chances of a good outcome diminish by each passing minute. Navigating the plethora of advanced imaging modalities means that treatment in some cases can be inefficaciously delayed. Time delays and individually based non-programmed decision making can prove challenging for clinicians. Visual aids can assist such decision making aimed at simplifying the use of advanced imaging. Flow charts are one such visual tool that can expedite treatment in this setting. A systematic review of existing literature around imaging modalities based on site of occlusion and time from onset can be used to aid decision making; a more program-based thought process. The use of an acute reperfusion flow chart helping navigate the myriad of imaging modalities can aid the effective treatment of patients.

## Introduction

Reperfusion therapy using intravenous thrombolysis (IVT) or endovascular treatment (EVT) is the gold standard of treatment for acute ischemic stroke (AIS) (1). Treatment effect with both modalities is not only highly time dependent but also has lower complication rates if expedited early (2–5).

Whether and how fast a patient with AIS receives treatment depends on several factors including system-level and physician-level factors (6). System-level factors include the availability of ambulances, local geography, advanced imaging, and staffing resources at nearby hospitals. At the physician level, there are two conceptual ways of making treatment decisions: programmed (automated: routine and can be made using a systematic approach) or non-programmed (unique: individual and requiring thought-based analysis) (7). The latter is time consuming and especially precarious in a setting where every minute counts (8). This is further compounded in stroke care where adherence to guidelines is variable (9). The decision making process is further complicated by access to advanced imaging. Where access to advanced imaging is a limiting factor transfer to the nearest comprehensive stroke center (CSC) should be considered. The decision to transfer a patient to the nearest CSC should be made in a timely fashion and made in the appropriate setting (10). Decision making now is therefore more complicated than ever and more time consuming.

The first EVT trials did not specify their imaging protocols in much detail; in fact, some did not even require vascular imaging (11–13). This heterogeneity in imaging, and subsequent variability in patient selection, was probably one of the reasons for their failure to show the benefit of EVT. Thus, subsequent randomized controlled trials (RCTs) for EVT had stricter, more homogeneous, yet relatively simple inclusion criteria that allowed for fast programmed decisions (14). These programmed decisions revolved around patient age, time from onset, and plain computed tomography (CT) findings. The same decision making pattern applied to patient selection for the early IVT trials. These trials only included patients based on age and time from symptom onset (15, 16). Using strict criteria and simple processes, decision making was more programmed but resulted in many patients being potentially excluded from treatment.

Recent data suggest that a greater number of patients can benefit from reperfusion treatment using a more individualized approach, by selecting patients using advanced imaging both for IVT and EVT (17–20). This has resulted in an update in guidelines, which now recommend the use of alteplase up to 9 h after the onset of stroke symptoms, given hypoperfusion-core mismatch on advanced imaging (21). EVT is reasonable in patients up to 24 h after symptom onset if advanced imaging criteria are met (1, 22). Some studies even indicate that selected patients meeting certain advanced imaging criteria might benefit from EVT more than 24 h after symptom onset (23). The selection process for reperfusion treatment in the late time window involves more and more non-programmed decision making based on a variety of relative variables instead of absolute cut-offs, and while this allows us to treat more patients, it also represents a challenge and can be confusing at times, particularly for less experienced physicians (7).

Herein, we review the available imaging modalities in AIS as relevant to reperfusion therapy and describe EVT and IVT decision making using different imaging modalities. We also provide a visual aid in the form of a flow chart to aid AIS decision making for acute reperfusion in clinical practice.

## Acute Ischemic Stroke and Imaging

Ischemic stroke symptoms are caused by a focal reduction in cerebral blood flow due to a vessel occlusion. Initially, the reduction of blood flow results in oligemia but upon worsening leads to ischemia followed by infarction if normal blood flow cannot be restored (24). Vessel occlusions in the setting of AIS can be broadly divided into large vessel occlusions (LVO), medium vessel occlusions (MeVO), and small vessel occlusions. An LVO is defined as an occlusion of the terminal intracranial carotid artery (ICA) and/or M1 segment of the middle cerebral artery (MCA) (14). A MeVO is defined as an occlusion of the M2 or M3 segment of the MCA, A2, or A3 segment of the anterior cerebral artery (ACA) and P2 or P3 segment of the posterior cerebral artery (PCA) (25).

LVO is currently the only vessel occlusion for which level 1A evidence exists demonstrating a clear benefit of EVT (1). These occlusions are unlikely to recanalize with intravenous thrombolysis alone, although current guidelines recommend IVT in addition to EVT where possible (26). Observational data suggest a beneficial effect of EVT for MeVO; however, data from RCTs are lacking (27).

In the early time window, defined as 4.5 h for IVT and 6 h for EVT, decision making is more programmed and treatment can be initiated on simplified imaging (plain CT and CT angiography). Outside the early time window, LVO and MeVO patients can be selected for both IVT and EVT based on advanced imaging (“tissue-window”). The goal of these advanced imaging methods is to classify tissue as irreversibly damaged (infarct core) or salvageable tissue (penumbra). Theoretically, patients with no salvageable tissue would not benefit from treatment and thus should not be unnecessarily exposed to treatment risks.

However, current advanced imaging methods do not allow us to accurately estimate infarct core and penumbra. This has casted doubt upon the validity of the term “infarct core” and led some authors to suggest the use of a more descriptive term, “severely ischemic tissue with unknown viability [SIT-uV]” (28). The limitations of imaging are important to bear in mind when relying on these methods for infarct core and penumbra estimation and treatment decision making.

## Computed Tomography

### Ruling Out Intracranial Hemorrhage and Identifying Early Ischemic Changes

Non-contrast head CT (NCCT) or plain CT is the most basic type of CT imaging and the first part of any CT-based AIS imaging protocol. Plain CT allows us to rule out intracranial hemorrhage, which constitutes a contraindication for IVT and EVT. Acute hemorrhagic stroke typically presents with hyperdense intraparenchymal foci of blood. NCCT also allows for a rough estimate of ischemia, which manifests as early ischemic changes: loss of gray-white matter differentiation, parenchymal swelling, and subtle parenchymal hypodensity. The Alberta Stroke Program Early CT Score (ASPECTS; aspectsinstroke.com) is a 10-point binary NCCT score that can be used to assess early ischemic changes in the middle cerebral artery territory. It divides the middle cerebral artery territory in 10 regions—seven at the ganglionic level and three at the supra-ganglionic level. A region is either scored as “affected” if early ischemic changes are present or “not affected” if no early ischemic changes are present in the region. For each affected region, one point is subtracted from an initial value of 10 points. Thus, 10 is the highest ASPECT score, indicating no ischemic changes in any of the territories, and 0 is the lowest possible score, indicating ischemic changes in all middle cerebral artery territories. A modified ASPECTS score for posterior circulation strokes (pcASPECTS) has also been described (29). If no intracranial hemorrhage and no extensive early ischemic changes are present, IVT can be administered, even before vascular imaging is performed. Although hyperdensity of an arterial vessel on NCCT, so-called “hyperdense vessel sign,” can indicate a vessel occlusion, definitive identification of the occluded blood vessel requires vascular imaging.

### Identifying Vessel Occlusion

**Figure 1:**
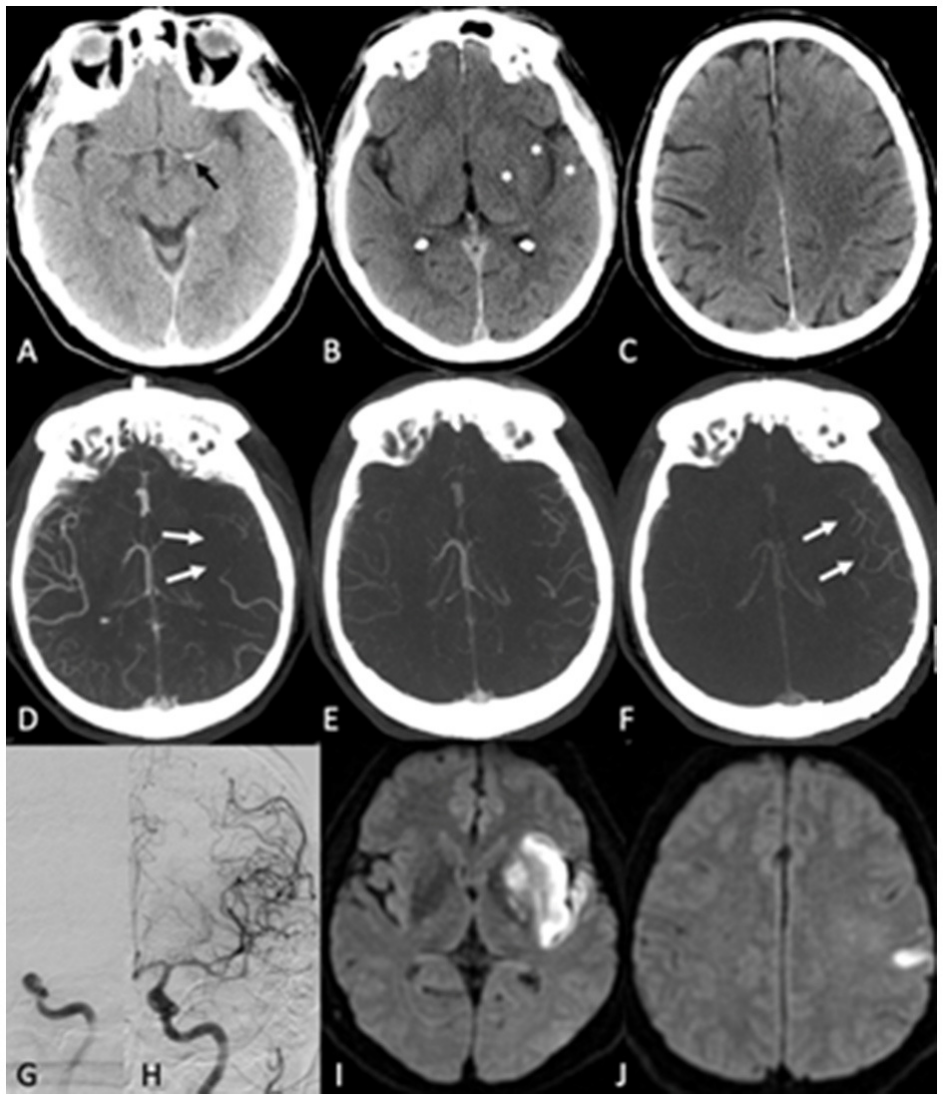
Acute ischemic stroke due to a terminal internal carotid artery occlusion. **(A,B)** show the initial Plain CT. A hyperdense vessel sign [black arrow in **(A)**] is seen, and there is loss of gray-white matter differentiation in the left insula, lentiform nucleus and M2 region [asterisks in **(B)**], corresponding to an ASPECTS score of 7. No ischemic changes are seen at the supraganglionic level **(C)**. Multiphase CTA shows lack of collateral filling in the first (arterial) phase [white arrows in **(D)**]. However, the pial arterial collaterals fill eventually in the second (peak-venous) phase **(E)**. There is a slight delay in washout in the third phase **(F)**. Intravenous thrombolysis was administered between Plain CT and mCTA, and the patient was treated with EVT after mCTA has been completed. **(G)** shows the initial digital subtraction angiography run with the occlusion. **(H)** shows the last intracranial angiography run with complete recanalization [modified thrombolysis in cerebral infarction (mTICI 3)]. On follow-up diffusion-weighted MRI at 24 h, an infarct of moderate size in the left insula, lentiform nucleus, and M2 region was seen **(I)**, corresponding to the areas with early ischemic changes in the initial plain CT. There was also a small infarct in the left M5 region **(J)** that was not noted on the baseline plain CT.

CT angiography (CTA), either single-phase or multiphase, is used to identify the presence of an LVO or a MeVO. In a single-phase CTA, a single arch-to-vertex angiography is obtained after contrast injection to depict the pre- and intra- cerebral vessels. In multiphase CTA (mCTA), two additional scans, following a single-phase examination, are obtained during the peak-venous and the late-venous phase (30). These last two scans image only the intracranial vessels (skull base to vertex) and are obtained using the same bolus contrast dose. Single-phase CTA has good sensitivity and specificity for LVO detection, with high inter-rater reliability (31). However, accuracy for MeVO detection is much lower, with up to a third of MeVOs being missed on single-phase CTA (32). Multiphase CTA can increase accuracy for MeVO detection by visualizing delayed filling and washout of pial arterial collaterals in the ischemic tissue (Figure 1) (33).

### Estimating the Infarct Core and Viable Penumbra

**Figure 2:**
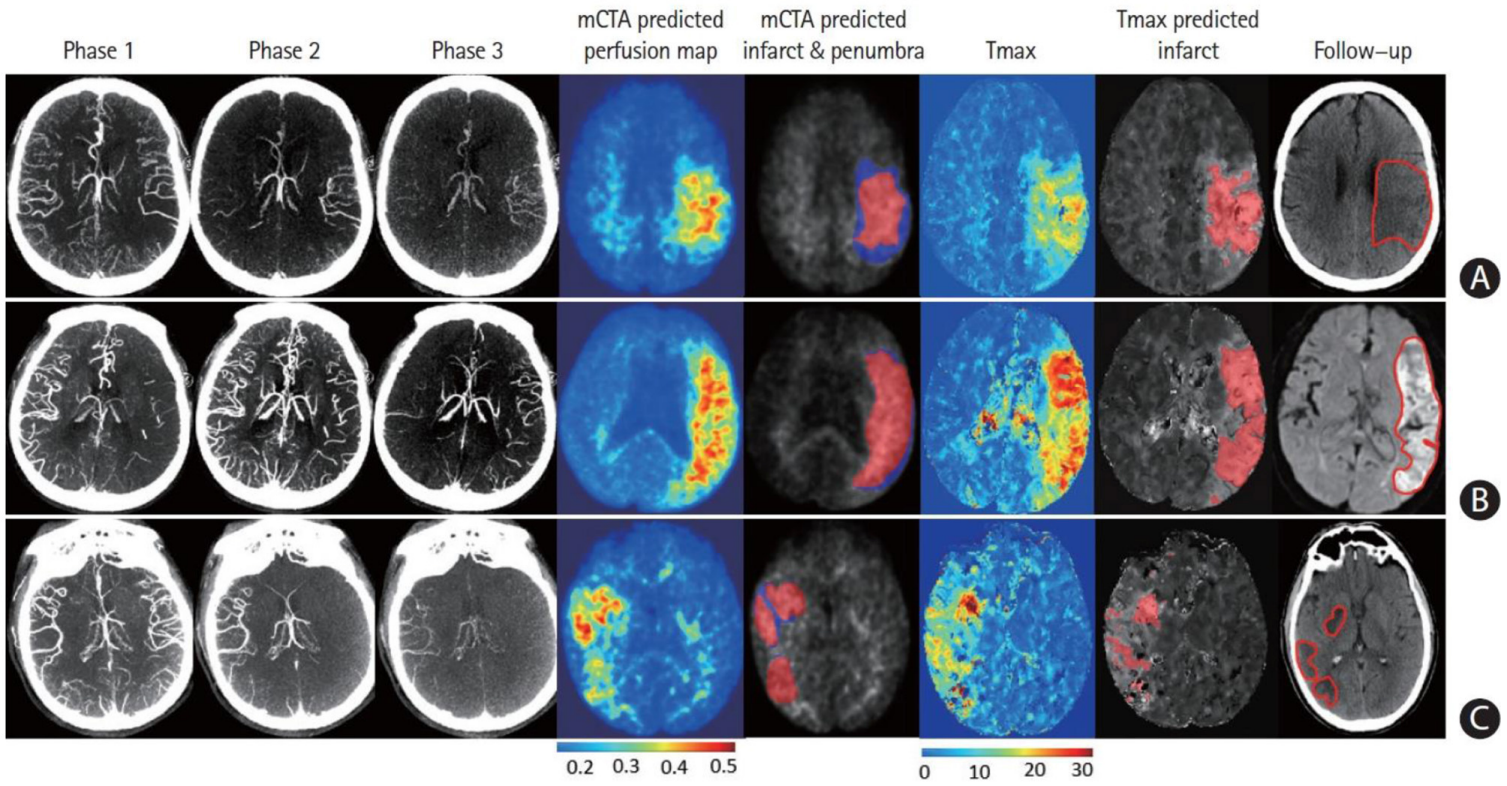
Multiphase computed tomographic angiography (mCTA) predicted an infarct map compared to a computed tomographic perfusion (CTP) time-dependent Tmax threshold map when compared to a follow-up infarct. **(A)** Patient who achieved reperfusion (mTICI 2b); **(B)** patient who did not achieve reperfusion; and **(C)** patient who achieved complete reperfusion with EVT. Columns: mCTA phase 1–3, mCTA predicted perfusion maps, mCTA predicted core (red in column 5) and penumbra (blue in column 5) overlaid on the mCTA predicted perfusion map, CTP Tmax maps, CTP time-dependent Tmax threshold predicted infarct, infarct contoured in follow-up imaging, respectively. The penumbra is shown as affected tissue from the penumbra model minus affected tissue from the core model. [Reprinted with permission from Journal of Stroke, 2021 (36)].

mCTA can be used to assess the infarct core both qualitatively and quantitatively. The time-resolved depiction of the collaterals allows for semiquantitative grading (30). Collateral circulation determined by such semiquantitative grading is an independent predictor of outcome following treatment with alteplase (34), and patients with good collaterals on baseline mCTA are more likely to benefit from EVT (35). Collateral grading on mCTA has a good interrater reliability, covers the whole brain, is robust against patient motion, and requires no post-processing. The use of machine-learning algorithms allows for derivation of tissue-level perfusion maps from mCTA that can be used to generate perfusion color maps and estimate core and penumbra volumes (Figure 2). Extracranial stenoses and poor cardiac output, however, could lead to underestimation of collaterals both in the qualitative and quantitative analysis.

In some ways, CT perfusion (CTP) can be seen as an extension of mCTA: the principle is the same, but time resolution is higher since 30–90 additional phases (as opposed to only two additional phases in mCTA) are obtained. After injection of an iodinated contrast bolus, a slab of 8–16 cm (depending on the scanner's detector width) is continuously imaged for 45–90 s. Several perfusion parameters, including cerebral blood volume (CBV), cerebral blood flow (CBF), mean transit time (MTT), and time to peak enhancement (TTP) are then calculated using a deconvolution algorithm and displayed using axial color maps, similar to mCTA tissue level perfusion maps (Figure 2). Decreased CBV is commonly used as a surrogate for ischemic core, whereas in penumbra, CBV is thought to be preserved. CTP was used for patient selection in some EVT trials in the early time window (37), and the DAWN and DEFUSE-3 trials have proven the benefit of EVT in patients with small CTP core and core-penumbra mismatch presenting between 6 and 24 h from symptom onset (17, 18). There is some evidence to suggest that patients fulfilling these CTP criteria can benefit from EVT even if they present beyond 24 h (23). The color-coded format of CTP and quantitative mCTA maps is easy to interpret for readers with little experience and can help to identify the occlusion location. However, one has to bear in mind that CTP is subject to technical failures in up to as many as one-third of patients (38). A recent publication highlights the fact that CTP often overestimates infarct core volumes (39). Furthermore, some studies suggest that CTP does not actually improve treatment decision making beyond mere occlusion detection (38).

### Posterior Circulation Stroke

NCCT is less sensitive for detecting ischemic stroke in the posterior fossa (sensitivity 41.8% CI 30.1–54.4) (40). The low sensitivity can be explained by several factors including the extensive beam-hardening bony artifacts in the posterior fossa, as well as the relative delay for strokes to appear on imaging in the white matter relative to the gray matter (41, 42).

## Magnetic Resonance Imaging

### Identifying Vessel Occlusion

Magnetic resonance imaging (MRI) sequences that are used to assess the vessels of the head and neck can be divided into non-contrast techniques [time of flight (TOF) MRA] and contrast-enhanced (CE) techniques (CE-MRA) (43). CE-MRA relies on injection of intra-arterial gadolinium-based contrast agents to depict the cervical and intracranial blood vessels, in a fashion similar to CTA. TOF-MRA, on the other hand, is a gradient echo sequence showing flow in vessels without the use of gadolinium contrast and can be acquired as 2D slabs or 3D volumes (44).

CE-MRA offers better diagnostic accuracy than TOF-MRA in acute ischemic stroke, being more accurate for identifying occlusion location (45, 46). However, CE-MRA has a lower diagnostic accuracy as compared to CTA: with a sensitivity of 80–92% and a specificity of 85–98% (47–49). Additionally, the time to achieve optimal arterial enhancement and acquisition times are longer as opposed to CTA, and the examination cannot be repeated with good quality until the intravascular gadolinium contrast is cleared (44).

TOF-MRA offers an alternative way to image intracerebral vessels in patients with contraindications to contrast agents. Furthermore, imaging can be repeated instantly as contrast wash out is not an issue (50). The sequence is however time-consuming and cannot accurately depict the extracranial vessels (51, 52). It is also more susceptible to motion artifacts because the signal is generated by directional blood flow (53).

### Estimating the Infarct Core and Viable Penumbra

Diffusion-weighted Imaging (DWI) detects hyperacute and acute ischemic lesions with a high sensitivity and specificity, with sensitivities ranging from 88 to 100% and specificities ranging from 86 to 100% (54–57). Acute cerebral ischemia causes cytotoxic edema mediated by a decrease in extracellular space and restricted diffusion of water molecules, which is reflected by an immediate decline in the applied diffusion coefficient (ADC). Typically, an ADC threshold of <620 μm/s is used by automated software to identify tissue with severely restricted movement of water (58). This correlates with tissue that is at risk of being irreversibly injured (59). Areas with reduced ADC appear hyperintense on DWI. DWI lesions are, in many cases, seen just minutes after the onset of symptoms (60, 61). The initial DWI lesion is thought to represent tissue at risk and correlates well with end infarct volume if no intervention is performed (44, 62, 63). Although DWI is far superior to plain CT at detecting hyperacute and acute ischemic change (47, 57), around 6% of AIS patients do not have any visible DWI lesions (64). Furthermore, DWI-reversible lesions have also been reported, particularly in the posterior circulation (31).

Subacute infarctions are characterized by the subsequent development of vasogenic edema, which is visualized as a hyperintense signal on T2-weighted fluid attenuated inversion recovery (FLAIR) MRI (65). FLAIR hyperintensity usually develops between 3 and 6 h after the onset of symptoms (60, 66–68). On rare occasions, however, the hyperintensity does not develop until up to 12 h after the onset of symptoms (69). The intensity of the signal can be used to temporally quantify symptom onset (70, 71). In one study, the positive predictive value of FLAIR hyperintensity increased from 0.87 to 1.00 when minor strokes, lacunar infarctions, and infratentorial strokes were excluded (66). Studies published as early as 1997 have shown that FLAIR hyperintensity is more clearly visible in cases of cortical supra-tentorial stroke (72).

Patients with a DWI-FLAIR mismatch, an acute ischemic lesion that is hyperintense on DWI but without any visible FLAIR correlation, are likely to be within a 3-h time window from symptom onset (19, 60). This has been implemented in clinical practice to assess the feasibility of IVT in patients where symptom onset is unknown, or the patient has awoken with stroke symptoms.

Perfusion weighted imaging (PWI) is used to map regional CBF, depicting hemodynamic conditions at the microvascular level, identical to CTP depiction. PWI can be obtained using either exogenous gadolinium contrast or endogenous contrast labeling [arterial spin labeling (ASL)]. For the most part, PWI in AIS is performed using an exogenous contrast agent (DSC—dynamic susceptibility contrast) (44). Following the intravenous administration of contrast, CBF, CBV, and MTT are calculated. These changes are depicted as color-coded maps as with CTP and mCTA tissue level perfusion maps (47). Using these parameters various thresholds for tissue viability for have been hypothesized. Most definitions of infarct core use a Tmax >6 s as threshold (73), while tissue with a Tmax <6 s is defined as oligemic tissue that is potentially salvageable. However, PWI alone cannot accurately distinguish between oligemia, penumbra, and core (47). The PWI-DWI mismatch, conceptually the same as the DWI-FLAIR mismatch, has been used to identify viable penumbra in the setting of reperfusion therapy (74–76).

### CT vs. MRI-Based Imaging in AIS

The biggest advantage of MRI-based imaging is that DWI shows hyperacute and acute infarcts with higher sensitivity and very early on (minutes after symptom onset) as compared to plain CT.

With respect to vascular imaging, CTA has a much higher sensitivity and specificity for the detection of vessel occlusions when compared to MRA (both CE-MRA and TOF-MRA). Additional advantages of CTA are its shorter acquisition time, tolerability of patient motion artifacts, and the option to acquire two additional phases for collateral assessment (mCTA). Furthermore, as opposed to TOF-MRA, the extracranial and intracranial arterial vasculature can be imaged simultaneously. The risk of contrast-induced renal damage and allergic reactions exist for both CE-MRA and CTA, but these complications more often associated with the use of iodinated contrast in CTA.

As for perfusion imaging, both PWI and CTP are subject to a significant technical failure rate and are associated with contrast related complications. Furthermore, some studies suggest that perfusion imaging does not actually improve treatment decision making beyond mere occlusion detection (38).

In general, most centers use a CT-based imaging protocol due to the fewer contraindications, faster acquisition times, greater availability, and lower costs compared to MRI.

### Posterior Circulation Strokes

DWI is the preferred imaging modality to exclude ischemia in the posterior circulation (40, 42). It is however important to recognize that DWI can be normal in posterior circulation strokes, especially in the hyperacute phase. Posterior circulation strokes had a five times greater chance of being so-called DWI-negative up to 72 h after symptom onset (64). FLAIR has a low sensitivity and specificity when it comes to identifying stroke in the posterior circulation (66). Therefore, it is difficult to rely on DWI-FLAIR mismatch as a form of tissue-based imaging in posterior circulation strokes. Applying the Posterior Circulation Alberta Stroke Program Early Computed Tomography Score (pc-ASPECTS) to pre-intervention DWI has been proven to be a sensitive and specific tool for predicting clinical functional outcome (77).

## Discussion

Recent advancements in reperfusion treatment, especially in the late time window, have diluted the hard and fast time-based exclusion criteria from the early reperfusion trials. The decision making was of a programmed type and based on strict time cutoffs. The ever-expanding indications for reperfusion therapies in AIS have led to the introduction of advanced imaging and “tissue windows” replacing time windows. Reperfusion decision making has thus evolved into non-programmed decision making. Decision making is ultimately complicated by two opposing factors: the time sensitive nature of treatment effect and the need to gather tissue information through neuroimaging (18, 59, 78).

Advanced imaging (CTP, PWI, DWI-FLAIR mismatch imaging) complements the more basic CT and MRI modalities. The sole purpose of advanced modalities is to establish the existence of viable penumbra. It is however important to note that the penumbra is fading by the minute, and attempting to interpret advanced imaging in a non-programmed fashion often leads to further confusion (79), as it requires interpretation of several relative variables rather than relying on absolute time cut-offs. Prolonged treatment windows and increased availability of advanced imaging also means that physicians should be well-informed about the limitations of the various imaging modalities used in acute ischemic stroke.

**Figure 3:**
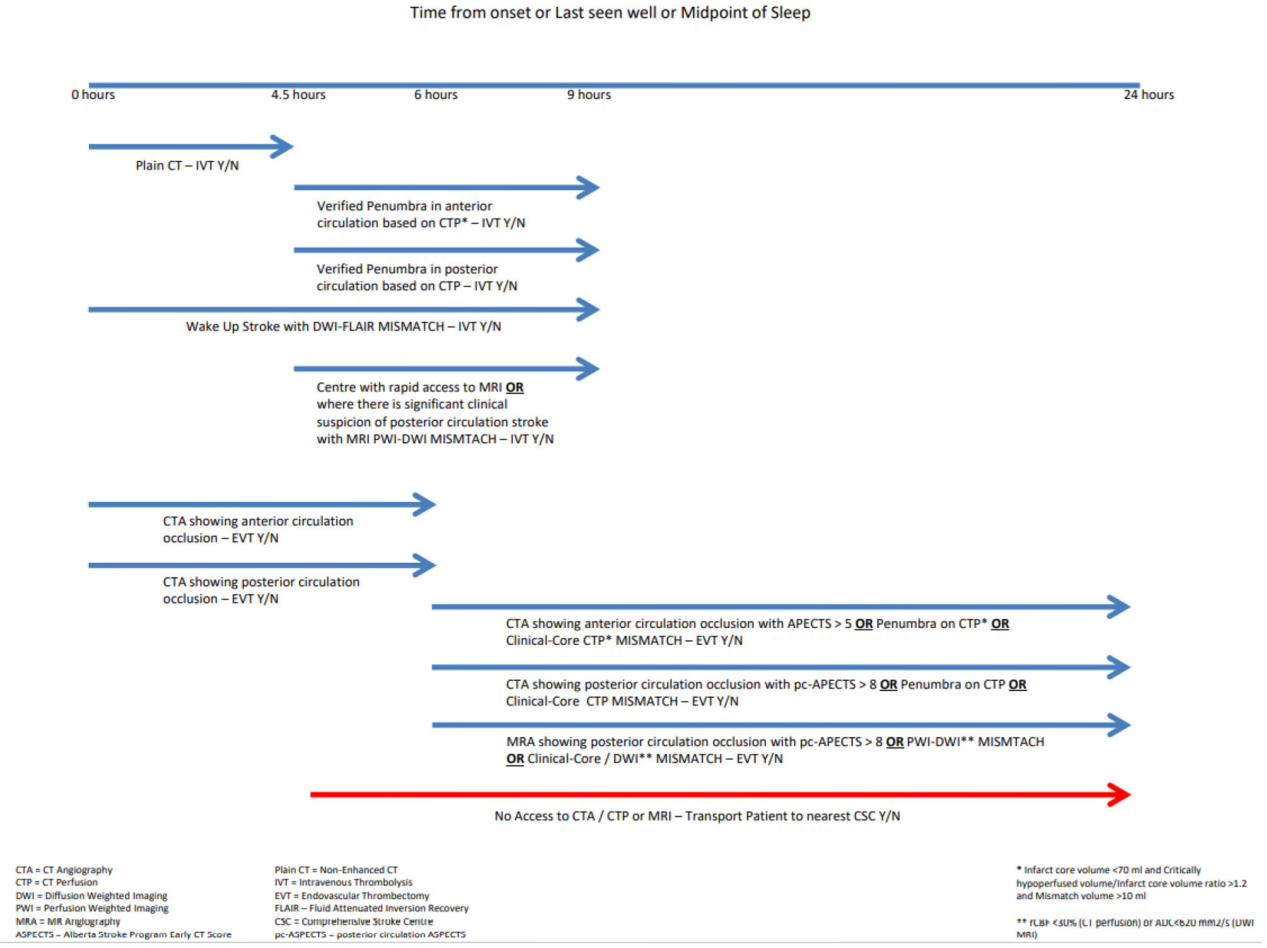
Flow chart for reperfusion decision making.

A simplification of imaging strategies is warranted, a program-based solution. The most pivotal piece of information for clinicians in this setting is time of onset. Establishing probable onset time through available information from the patient or a stroke witness is key. It is also important to note that imaging is only of limited value to establish time of onset, since the DWI-FLAIR mismatch has some variability and can falsely preclude treatment (78, 80). Therefore, clinical assessment of symptom onset is crucial to assist in the program-based decision making process. Once onset time or time since the patient was last seen well has been established, tissue-based imaging can be utilized to visualize penumbra (Figure 3).

In the early time window (0–6 h), less is more as far as imaging is concerned. Using plain CT to verify the lack of extensive early ischemic changes and CTA to verify an LVO or MeVO before going directly to the angiography suite is the most efficient strategy. In the late time window (6–24 h from last known well) the plethora of tissue-based imaging modalities comes into play. Selecting patients in the late time window is tissue-based rather than time-based (81). Of note, the 6-h cutoff is based on the inclusion criteria of early EVT RCTs and does not in any way reflect the underlying physiological variability neuronal loss (8).

Imaging modalities for tissue-based patient selection include CT-based (CTP) and or MRI based strategies (PWI), as well as the DWI-FLAIR mismatch. The type of tissue-based imaging used should be chosen based upon site of occlusion in the late window. In anterior circulation strokes, both CT- and MRI-based perfusion strategies have been successfully used to identify penumbra (59). A preference for use of these modalities ultimately comes down to traditions of practice and availability at individual centers. This has been well-reflected in the late window EVT RCTs in the anterior circulation, in which both CT-based and MRI-based protocols were successfully used (18, 82). It is worth bearing in mind that MRI tissue-based imaging is more time consuming, more susceptible to motion artifacts, and has a greater number of contraindications than CT imaging. The use of CT imaging is therefore more congruent with program-based decision making (83).

In the setting of posterior circulation strokes, PWI-DWI mismatch seems to be a more reliable marker of viable penumbra than CTP findings (83). Thus, to aid program-based decision making in the late window, in posterior circulation strokes MRI PWI-DWI mismatch should be preferred where available.

Figure 3 provides a visual aid to increase accuracy and timeliness of reperfusion decision making based on tissue imaging and up-to-date guidelines (84, 85). Focusing on onset time and site of occlusion helps decision making and simplifies the acute treatment setting. The figure also maintains focus on imaging where transporting the patient to a CSC should not be forgotten.

Recent ESO recommendations regarding the use of IVT in late window thrombolysis also highlight the importance of tissue-based imaging. IVT has been traditionally administered within 4.5 h of symptom onset, but there is now a strong recommendation that alteplase should be considered between 4.5 and 9 h of symptom onset where viable penumbra is detected on advanced imaging (21). The updated ESO guidelines recommend the use of advanced imaging to establish a penumbra core mismatch ratio >1.2 with an infarct core of <70 ml. The recommendations do not favor the use of MRI over CT-based perfusion imaging, and each center should build on the use of the modality already previously established in treatment routines. In program-based decision making, however, simplifying algorithms reduces treatment times and expedites correct treatment (4). Thus, adhering to CTP only in anterior circulation strokes and MRI PWI-DWI mismatch in posterior circulation strokes is more congruent with program-based decision making leading to improved efficacy.

With the updated guidelines reinforcing the need for advanced imaging, it is crucial to consider the availability of advanced imaging at each treatment center. Transport of patients from a primary stroke center (PSC) to a comprehensive stroke center (CSC) is warranted where there is limited or no access to advanced imaging in cases where treatment can be initiated.

Extended reperfusion time windows and the growing availability of advanced imaging can become a labyrinth of pitfalls and missed opportunities for many stroke physicians, and patients, for that matter. A robust understanding of the imaging modalities and their limitations is paramount if timely decision making is to be expedited. The use of visual aids in the acute treatment setting can help revert back to a program-based decision making strategy for acute ischemic stroke patients, thus securing efficacious treatment for many more patients.

## Author Contributions

MG, RA, SA, MK, and JO contributed to the writing of the manuscript. MS, ES, and MK contributed to the editing of the manuscript. MS, ES, and RA contributed to the conceptualization of the manuscript. All authors contributed to the article and approved the submitted version.

## Conflict of Interest

The authors declare that the research was conducted in the absence of any commercial or financial relationships that could be construed as a potential conflict of interest.

## Publisher's Note

All claims expressed in this article are solely those of the authors and do not necessarily represent those of their affiliated organizations, or those of the publisher, the editors and the reviewers. Any product that may be evaluated in this article, or claim that may be made by its manufacturer, is not guaranteed or endorsed by the publisher.
